# Efficiency and Mechanism of Surface Reinforcement for Recycled Coarse Aggregates via Magnesium Phosphate Cement

**DOI:** 10.3390/ma17010122

**Published:** 2023-12-26

**Authors:** Siyao Wang, Jingtao Hu, Zhiyuan Sun, Yuan Gao, Xiao Yan, Xiang Xue

**Affiliations:** 1 School of Transportation and Civil Engineering, Nantong University, Nantong 226019, China; wangsiyao@ntu.edu.cn (S.W.); jingtaohu2003@163.com (J.H.); 15937747640@163.com (Z.S.); y.gao@ntu.edu.cn (Y.G.); 2 Department of Geotechnical Engineering, College of Civil Engineering, Tongji University, Shanghai 200092, China; 3 School of Civil Engineering, Chongqing University, Chongqing 400044, China

**Keywords:** recycled aggregate concrete, magnesium phosphate cement, mechanical properties, durability, microstructure

## Abstract

Recycled aggregate concrete (RAC) exhibits inferior mechanical and durability properties owing to the deterioration of the recycled coarse aggregate (RCA) surface quality. To improve the surface properties of RCA, the reinforcement efficiency of RAC, and the maneuverability of the surface treatment method, this study used magnesium phosphate cement (MPC), a clinker-free low-carbon cement with excellent bonding properties, to precoat RCA under three-day pre-conditioning. Moreover, variable amounts of fly ash (FA) or granulated blast furnace slag (GBFS) were utilized to partly substitute MPC to enhance the compressive strength and chloride ion penetration resistance. Subsequently, FA–MPC and GBFS–MPC hybrid slurries with the best comprehensive performance were selected to coat the RCA for optimal reinforcement. The crushing value and water absorption of RCA, as well as the mechanical strengths and durability of RAC, were investigated, and microstructures around interfaces were studied via BSE-EDS and microhardness analysis to reveal the strengthening mechanism. The results indicated that the comprehensive property of strengthening paste was enhanced significantly through substituting MPC with 10% FA or GBFS. Surface coating resulted in a maximum reduction of 8.15% in the crushing value, while the water absorption barely changed. In addition, modified RAC outperformed untreated RAC regarding compressive strength, splitting tensile strength, and chloride ion penetration resistance with maximum optimization efficiencies of 31.58%, 49.75%, and 43.11%, respectively. It was also evidenced that the improved MPC paste properties enhanced the performance of modified RAC. Microanalysis revealed that MPC pastes exhibited an excellent bond with RCA or new mortar, and the newly formed interfacial transition zone between MPC and the fresh mortar exhibited a dense microstructure and outstanding micro-mechanical properties supported with an increase in the average microhardness value of 30.2–33.4%. Therefore, MPC pastes incorporating an appropriate mineral admixture have enormous potential to be utilized as effective RCA surface treatment materials and improve the operability of RCA application in practice.

## 1. Introduction

Making recycled aggregate concrete (RAC) [1,2,3] is a very efficient approach to using construction and demolition waste [4,5] for resource-saving and environmental protection disposal, in which the natural coarse aggregate (NCA) is partially or wholly replaced with recycled coarse aggregate (RCA) [6,7,8]. Researchers have shown that RAC’s mechanical properties and durability decreased with increased replacement by RCA [9,10,11], which hinders RAC’s sustainable development and application [12,13,14]. The primary reason for this phenomenon is the high porosity and water absorption of the old mortar attached to the RCA surface [15], causing the new interfacial transition zone (ITZ) to be more porous and the interfacial bond strength between the RCA and new mortar to be weaker.

Accordingly, to expand the application of RAC, many RCA surface treatments have been proposed to enhance the performance of RAC through improving the new ITZ [16]. These include soaking or surface precoating with polyvinyl alcohol (PVA) [17], silane polymers [18,19], sodium sulfate solutions [20], sodium silicate solutions [21], volcanic ash materials [22,23,24], cement, and other cementitious materials [25,26,27,28], as well as accelerated carbonation [29,30,31] and biological carbonate deposition [29,32,33]. However, these techniques might be hampered by a lack of durability from polymer compounds, a weak bond between the surface-coating paste and the RCA, and uncertainty about the efficacy of the carbonation treatment. To this end, Chen et al. [34] suggested a novel surface treatment technique in which magnesium phosphate cement (MPC) was used as a “bridge” between fresh concrete mortar and RCA. As a low-carbon green cementing material, MPC was generally acknowledged to have a high bonding strength with existing concrete, ranging from 77% to 120% higher than that of ordinary Portland cement [35], as well as high volume stability [36], and excellent durability for application in a diversity of complex environments [37,38]. Therefore, MPC can overcome the poor bonding strength of surface-coating pastes to RCA and effectively improve the mechanical properties of RAC [39,40,41]. Nevertheless, the modified RCA needed a long curing period for use and thus may delay the duration of construction of RAC application in practice [34]. Moreover, the effect of MPC on the long-term performance [42] of RAC lacks proof, which is likewise a primary concern in engineering applications. Thus, to enhance the operability of this treatment for engineering applications, the physical properties of MPC-modified RCA under short curing ages and the corresponding RAC’s mechanical performance and durability need to be further investigated. In addition, interfacial adhesion enhancement between MPC paste and RCA or new mortar, as well as improvement of the new ITZ, have not been thoroughly studied, which is crucial to reveal the enhancement mechanism of surface reinforcement by MPC paste.

Meanwhile, the performance of both RCA and RAC has been confirmed to exhibit a strong correlation with the fundamental property of strengthening pastes. For instance, the water absorption and crushing value of RCA were affected by the strengthening paste’s hardened strength and anti-permeability., The strength of RAC was likewise related to the mechanical strength and compactness of the strengthening paste [43,44,45]. According to studies, mineral admixtures have been frequently employed in MPC systems in appropriate dosages as cost-effective, ecologically friendly components that enhance MPC qualities. For example, utilizing the “ball effect”, micro-aggregation effect [46,47], and hydration-induced effect, fly ash (FA) can improve the later mechanical characteristics of FA–MPC [48,49,50]. Moreover, granulated blast furnace slag (GBFS) could improve the mechanical properties and durability of MPC due to the physical filling and the chemical reactions contributed by the presence of calcium components [51,52]. Accordingly, to acquire the optimum efficacy of surface coating, the mineral admixtures FA or GBFS could be incorporated into MPC materials, and the appropriate dosing amounts need to be investigated.

In this study, a series of experiments were carried out with the aim of revealing the reinforcement efficiency of RCA and RAC via surface treatment with MPC paste under a short pre-conditioning time, as well as the influence of MPC slurry properties on the reinforcement efficiency for RCA and RAC. Firstly, different surface-strengthening pastes were prepared with MPC supplemented by 0%, 5%, 10%, and 15% of FA or GBFS. Subsequently, the properties of surface-strengthening pastes, including compressive strength, chloride ion penetration resistance, and the synergistic mechanism of MPC with FA or GBFS, were examined. Based on the results obtained, the optimal FA or GBFS dosage can be selected, and the corresponding MPC blend pastes were utilized for the surface enhancement of RCA. Afterward, the enhancement effects of the surface coating on RCA and RAC were verified through testing the water absorption and crush value of RCA, as well as the mechanical strength and chlorine ion penetration resistance of RAC. Finally, the microstructural properties and elemental distributions of the bond interface and new ITZ were characterized through conducting backscattered electron and energy dispersive spectroscopy (BSE-EDS) measurements as well as microhardness tests to reveal the microscopic strengthening mechanism of various MPC pastes on RAC macroscopic properties. This study assists in improving the operability of RCA application in practice, promoting the production of high-quality RAC, and thus contributing to fostering sustainable development of the construction industry and yielding environmental benefits.

## 2. Materials and Methods

### 2.1. Major Raw Materials

In this study, dead-burned magnesia (MgO), ammonium dihydrogen phosphate (NH_4_H_2_PO_4_, abbreviated as ADP), borax (Na_2_B_4_O_7_·10H_2_O, abbreviated as B) as a retardant, and water were combined in precise ratios to create a pure MPC paste, referred to as S-0. MgO and ADP were purchased from Liaoning Yangyang High Tech Materials Co., Ltd. in Yingkou City, and B was purchased from Zhiyuan Chemical Reagent Co., Ltd. in Tianjin. Additionally, 5%, 10%, and 15% of the mass of MgO were replaced with FA or GBFS to create mineral admixture–MPC pastes. FA and GBFS were purchased from Ningdong Thermal Power Co., Ltd. in Yinchuan City and Rongchangsheng Environmental Protection Materials Co., Ltd. in Zhengzhou City, respectively. The chemical compositions of FA, GBFS, and MgO used in this study were determined via X-ray fluorescence (XRF) oxide analysis, and the results are presented in Table 1. Purities of the industrial-grade ADP and B were above 98% and 99.5%, respectively. The particle size distributions of FA, GBFS, and MgO were examined using a laser particle size analyzer, and the average particle sizes were approximately 9 μm, 7 μm, and 12 μm for FA, GBFS, and MgO, respectively.

**Figure 1:**
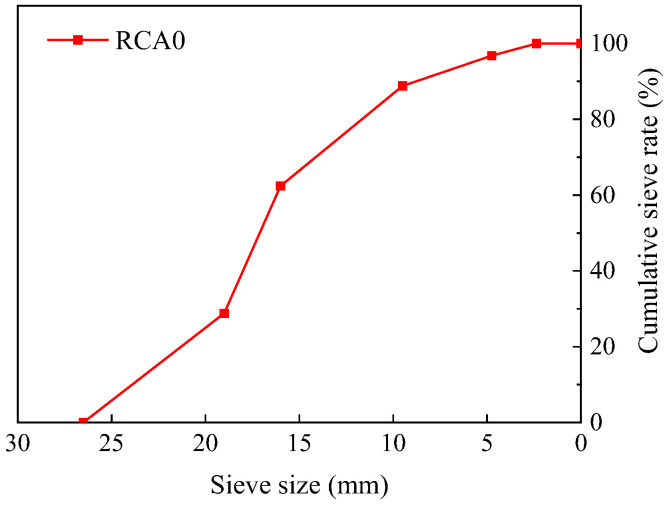
Particle size distribution of RCA0.

Using an experimental jaw crusher, the untreated RCA used in this study was produced from original concrete with a compressive strength of approximately 35 MPa and labeled as RCA0. Figure 1 displays the gradation information of RCA0 obtained from the sieving method. According to Chinese Standard GB/T 14685-2022 [53], the accuracy of the sieving method could be guaranteed based on the sampling process. The sampling process was specified as follows: first, the sample was formed through randomly selecting aggregates of approximately equal mass from different portions of the aggregate heap; then, the sample was placed on a flat plate, mixed well under natural conditions, and piled up into a heap; afterward, the heap was divided into four equal portions along two diameters perpendicular to each other, and the two diagonal portions of the heap were re-mixed and piled up into a heap; the process was repeated until the amount of sample was reduced to that required for the test. The experiment also utilized natural river sand with a fineness modulus of 2.63 and water absorption of roughly 1.9%, as well as Portland cement (P.O. 42.5), with mechanical and physical parameters shown in Table 2. A water reducer was also included to improve the workability of the concrete.

### 2.2. Preparation Methods

#### 2.2.1. Surface-Strengthening Pastes

Different amounts of FA and GBFS were used to replace MgO to prepare blended MPC pastes. Table 3 shows the mixing ratio of each MPC paste required to modify 1000 kg RCA0. Precast MPC paste was prepared through blending the non-water components of the mixture materials based on the prescribed ratio first, then adding the corresponding amount of water and mixing for 60 s. After that, each type of precast MPC paste was cast in six 40 mm^3^ cubic molds and six cylindrical molds with a size of Φ100 × 50 mm^3^. Then, all specimens were demolded after 3 days and maintained at 20 ± 2 °C and 64 ± 2% RH for 28 days. The cylindrical specimens were used for the rapid chloride permeability test (RCPT). In addition, the cubic specimens were used for the compressive strength test, and approximately 10 mm^3^ pieces were cut from the fractured hardened blocks for BSE-EDS analysis. In detail, for microscopic test sample preparation, the slices were first soaked in ethanol for 24 h to halt cement hydration, then dried and embedded in epoxy resin with a cylindrical rubber mold measuring 20 mm in height and 25 mm in diameter, and finally, the samples were polished to create a smooth surface, dried, and stored in a vacuum chamber before testing.

#### 2.2.2. Surface-Reinforced RCA

The basic properties of strengthening pastes containing different amounts of FA or GBFS were tested regarding compressive strength and chloride ion penetration resistance. Based on the results, the most optimal FA or GBFS dosages leading to higher strength and lower chloride penetration were determined, and the corresponding blended MPC pastes were selected to prepare surface-reinforced RCA. Furthermore, S-0 was chosen as a comparison. It was expected that the physical properties of RCA, including water absorption and crushing value, could be improved through surface strengthening [44,45,54].

After preparation, the strengthening paste was immediately mixed and stirred with RCA0 for 5 min to precoat RCA0. Subsequently, the RCA was removed from the tank, and any excess MPC paste stuck to it was sieved away. Afterward, these treated RCA were exposed to the air with 64 ± 2% RH and 20 ± 2 °C for 3 days. A portion of the RCA was used for characterization tests, including water absorption and crushing value; another portion of the RCA was used to prepare RAC.

#### 2.2.3. Concrete

**Figure 2:**
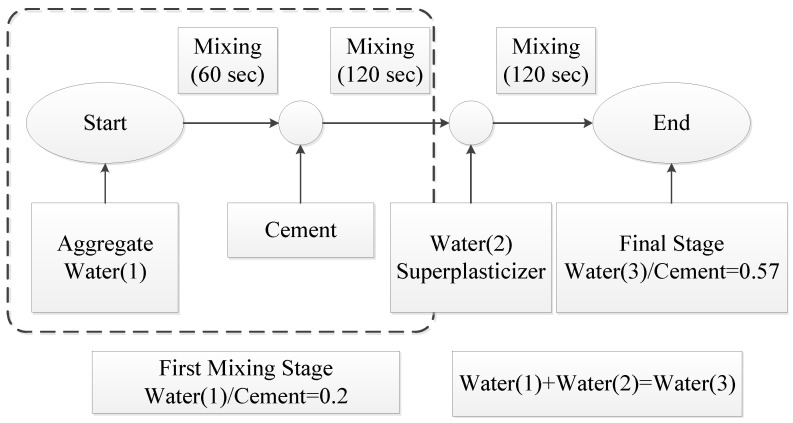
Double mixing method.

The original RCA0 and treated RCA obtained from Section 2.2.2 were utilized as coarse aggregate to produce concrete to obtain the best modification effect for high-quality RAC and the influence of the strengthening paste properties on the modification efficiency. Since the coated paste amount was negligible compared to the weight of RCA (approximately 2% to 3%) [43], based on a concrete strength grade of C30, the mix proportion for each concrete type was cement:water:sand:coarse aggregate:superplasticizer = 431:247:767:989:2. In order to get superior modification outcomes, this work adopted the double mixing method [55,56] to prepare modified RAC utilizing surface-treated RCA, and it has been demonstrated to reduce the water–to–cement ratio of the new ITZ thereby improving the interface zone, compressive strength, and chloride ion penetration resistance of concrete [55,57,58]. The specific mixing procedure, as shown in Figure 2, is as follows: first, a portion of the water (Water(1)) was added to the aggregates of each group and stirred for 60 s to obtain moist aggregates; then, cement was added and stirred for 120 s to coat the aggregate surfaces with a layer of low water–to–cement ratio cement slurry; finally, the remaining water (Water(2)) was added along with the superplasticizer used, and the fresh concrete was obtained through mixing for 120 s.

The mechanical strength and chloride ion penetration resistance of concrete were compared to understand the difference in the enhancement of RCA with various strengthening pastes. Each type of target concrete specimen consisted of six cubic specimens with dimensions of 100 mm^3^ and six cylindrical specimens with diameters of 100 ± 1 mm and heights of 50 ± 2 mm. All specimens were cured in a laboratory environment (25 ± 2 °C, 95 ± 2% RH) for 28 days. In order to prepare samples for microstructure analysis, slices were cut from the fractured hardened blocks obtained after the mechanical strength testing, whose surface included the desired testing areas containing the interface. Detailed procedures for sample preparation can be obtained from Section 2.2.1.

### 2.3. Test Methods

#### 2.3.1. Performance Testing of Strengthening Pastes

Compressive strength testing was conducted on the 2000 kN servo-hydraulic compressional testing machine according to GB/T 17671-2021 [59]. Moreover, RCPT was used to determine the resistance to chloride penetration of each group of strengthening paste. The procedure from specimen preparation to testing is detailed in the ASTM C1202-19 standard [60]. Furthermore, each group’s chloride ion permeability of the strengthening paste was qualitatively graded via the mean electrical flux.

Microscopic examination of different types of hardened paste was conducted using scanning electron microscopy (SEM, TESCAN MIRA LMS, Czech Republic) equipped with EDS (Oxford Xplore). BSE-EDS pictures were captured and utilized to investigate the strengthening mechanisms of FA and GBFS on the microstructure of hardened MPC pastes, thus providing more information on the effect of the paste’s properties on RAC performance. The imaging machine operated at a 15 mm working distance with a 15 kV voltage.

#### 2.3.2. Characterization Testing of RCA

(1) Water absorption

The water absorption of RCA was derived via the following equation:

(1) $$Water\;absorption\;ratio=\frac{wet\;weight-dry\;weight}{dry\;weight}*100\%$$

The wet weight and dry weight of aggregates could be measured based on Chinese Standard GB/T 14685-2022 [53].

(2) Crushing value

The crushing value tests of RCAs were carried out based on Chinese Standard GB/T 14685-2022 [53], and the crushing value could be calculated following the formula below:

(2) $$Curshing\;value=\frac{G_{2}}{G_{1}}*100\%$$

*G*_1_ and *G*_2_ were the total weight of aggregates and the weight of crushed aggregates finer than 2.36 mm, respectively.

#### 2.3.3. Macroscopic Properties Testing of Concrete

Each concrete group’s compressive and splitting tensile strengths were tested using three cubes, following the guidelines specified in GB/T 50081-2019 [61]. The RCPT was conducted to determine each concrete group’s chloride ion penetration resistance after 28 days of curing, and the evaluation was conducted following ASTM C1202-19 [60].

#### 2.3.4. Microscopic Characterization Testing of Interfaces

(1) BSE-EDS testing

To examine the microstructure and precise elemental distribution, BSE-EDS imaging was carried out on the bond interfaces between strengthening pastes and RCA0, as well as the new ITZ regions. This made it possible to disclose the bonding and strengthening mechanisms of the strengthening paste on RCA0 and the new ITZ. The preparation method of the BSE-EDS testing samples has been described in Section 2.2.1.

(2) New ITZ microhardness testing

**Figure 3:**
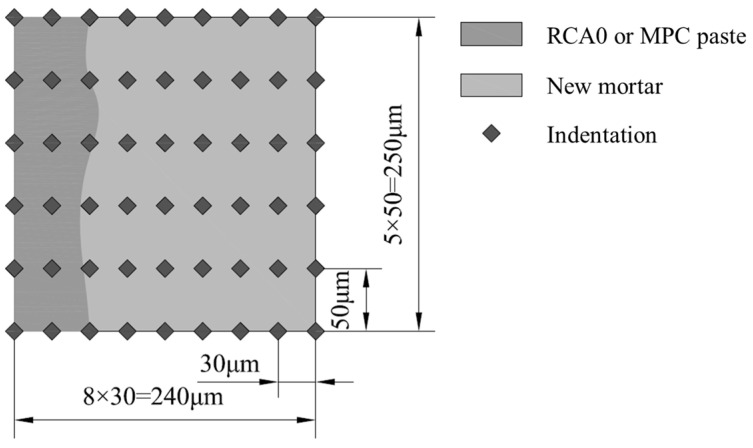
Indent area and corresponding indent matrix for new ITZ’s microhardness testing.

In addition to the microstructural composition, the microscopic mechanical properties of materials are also critical microstructural characteristics. Microhardness (Vickers hardness) has been used to understand the microscopic mechanical characteristics of RAC [62,63,64]. Therefore, to further validate the new ITZ performance improvement due to MPC modification, microhardness tests were performed on the regions containing the new ITZ in all RAC specimens, using a digital Vickers microhardness tester equipped with 40 measurement objectives and 10 magnification objectives (HV-1000BZ, Shanghai, China). As shown in Figure 3, the test region size was 240 μm × 250 μm, and a 9 × 6 indent points matrix was applied within the region. The samples employed for the microhardness test are detailed in Section 2.2.3. At least three areas were chosen randomly from two samples of each target concrete for testing. The two-dimensional microhardness distribution maps for each indent region were generated using the Surfer 13’s Contour map feature. The new ITZ’s boundaries were identified based on the color variations observed in the microhardness distribution maps. The average microhardness values of the new ITZ were determined using statistical analysis according to the microhardness values of each indent point within the boundaries.

## 3. Results and Discussion

### 3.1. Performance Characteristics of Strengthening Pastes

#### 3.1.1. Macroscopic Performance of Strengthening Pastes

Based on previous research, the modified RCA and RAC’s properties correlate firmly with the surface-strengthening paste’s performance, which can be strengthened through adding appropriate amounts of mineral admixtures. Therefore, this section compared the compressive strength and chloride ion penetration resistance of hardened MPC. On this basis, it was expected to select the best-performing FA-doped or GBFS-doped MPC paste for coating RCA.

**Figure 4:**
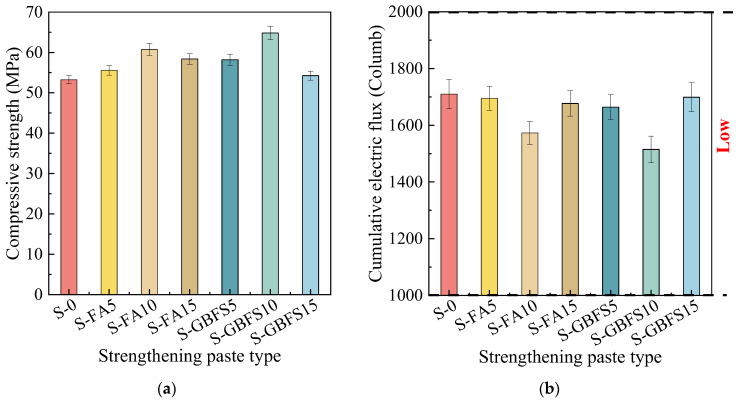
Different MPC pastes’ (**a**) compressive strengths and (**b**) cumulative electric fluxes.

The compressive strengths of prefabricated MPC pastes are shown in Figure 4a. It can be indicated that the compressive strengths of the blended MPC pastes were higher than that of S-0 without mineral admixture on the condition that the admixture of either FA or GBFS was 5%, 10%, and 15%. Moreover, it can be further seen that the compressive strengths of MPC pastes tended to increase and then decrease with increasing dosages of both mineral admixtures. That is, the optimal dosing for both FA and GBFS is 10%, and in that condition, the compressive strengths were 60.73 MPa and 64.82 MPa, and an increase of 7.49 MPa and 11.58 MPa in comparison with S-0 was exhibited, respectively. S-GBFS10 had the most significant gain among them.

Figure 4b displays the cumulative electrical fluxes that passed through all MPC pastes in six hours. It can be seen that the cumulative electrical flux for each type of MPC paste was relatively minimal, categorizing them as “low”. Their excellent resistance to chloride ion penetration can be attributed to the low water–to–cementitious material ratio and drying shrinkage of the MPC. Moreover, the graph demonstrates that the electrical fluxes of the MPC pastes exhibited the most significant diminution through adding 10% mineral admixtures. Compared to the S-0 paste at 1710C, S-FA10 and S-GBFS10 exhibited reductions of 137C and 195C, respectively. This indicates that adding additive FA or GBFS in the proper quantity dramatically improved the MPC paste’s density and impermeability. Combining these findings with those from the compressive strength test, it was quickly found that a better comprehensive performance could be obtained on the condition that FA or GBFS doping was 10%. The chief reason for this could be inferred as the mineral admixture in the right amount may play the role of physical filling and facilitate the secondary reaction, thus enhancing the microstructure of MPC paste, yet the excessive substitution of MgO with mineral admixture resulted in a decrease in the number of hydration products and thus led to the poor densification of MPC microstructure [65]. Hence, S-FA10 and S-GBFS10 were chosen to reinforce RCA for their excellent comprehensive performance. The strengthening mechanism of FA or GBFS for the mineral admixture–MPC system’s microstructure will be interpreted in detail in the following section.

#### 3.1.2. Enhancement Mechanisms for MPC via FA or GBFS

**Figure 5:**
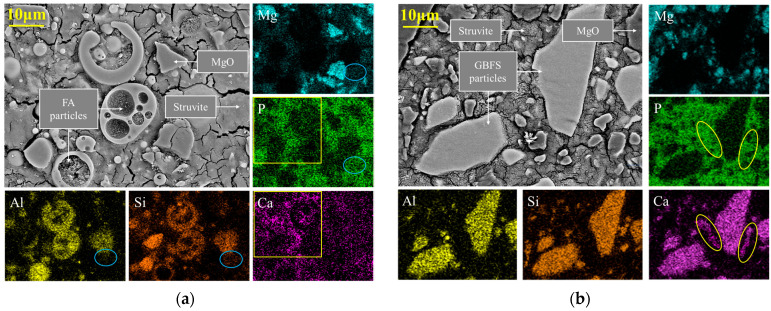
Typical BSE images and elemental maps of hardened (**a**) S-FA10 paste and (**b**) S-GBFS10 paste.

To explore the strengthening mechanism of both mineral admixtures on the macroscopic properties of MPC pastes, Figure 5a,b displays the BSE-EDS images of hardened S-FA10 and S-GBFS10 pastes at high magnification, respectively. It can be recognized that both hardened pastes exhibited the creation of the struvite phase, and unreacted MgO grains were detected throughout the matrix and appear to be the nucleation sites for struvite formation. It was evident from the reaction equation between MgO and ADP that there would be some solid volume expansion from MgO to struvite, resulting in a denser microstructure of the MPC paste. Due to dehydration under vacuum for examination, the embedded struvite particles in the polished parts seemed severely cracked. The spherical particles of various sizes in Figure 5a were FA particles. It can be seen that several medium-sized (10–20 μm) spherical particles were identified as surface depressions, indicating that the particles underwent partial reactions [66], whereas smaller particles with similar erosion depths on their surfaces may have undergone complete reactions or dissolution. In the EDS images of Figure 5a, the region where the elements P and Ca appeared to overlap significantly, marked with yellow wireframes, as well as the region where the elements Mg, P, Si, and Al seemed to coincide, marked with blue coils, also provided evidence of chemical reactions between the aluminosilicate FA particles and the other constituents in the MPC paste. The reaction products were speculated to be calcium phosphate, enstatite, and berlinite [41] based on recent evaluations [51,67]. In Figure 5b, the angular particles of various sizes rich in Ca, Al, and Si elements corresponded to the unreacted calcium aluminosilicate glassy portion in GBFS particles. The regions highlighted with the yellow coils in the elemental maps of Figure 5b indicated that the active calcium oxide in GBFS reacted with phosphate in the matrix, leading to the formation of calcium phosphate gel, which was consistent with the mechanism of GBFS being used as an adsorbent for phosphate removal in wastewater systems [68]. Therefore, it may be inferred that FA or GBFS will form a strong link with the surrounding hydration products and act as aggregates within the matrix due to their dissolution and subsequent reaction. Consequently, the MPC matrix’s integrity was improved, and its strength and permeability resistance were significantly boosted.

Furthermore, based on the percentages of calcium oxide and aluminosilicate components in the mineral admixtures, as well as the test results of compressive strength and chloride ion penetration resistance, we prefer to believe that the calcium oxide-related reaction dominated the synergistic effect between MPC and FA or GBFS. This finding aligned with the conclusion drawn in the reference [52].

### 3.2. Performance Characteristics of RCA

**Figure 6:**
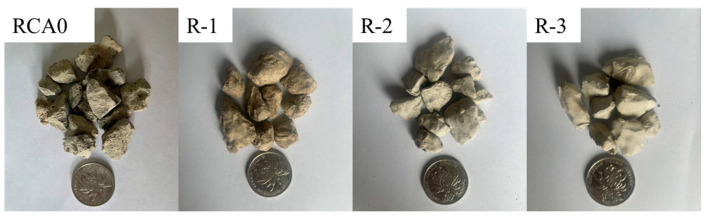
The modified RCA and RCA0.

**Figure 7:**
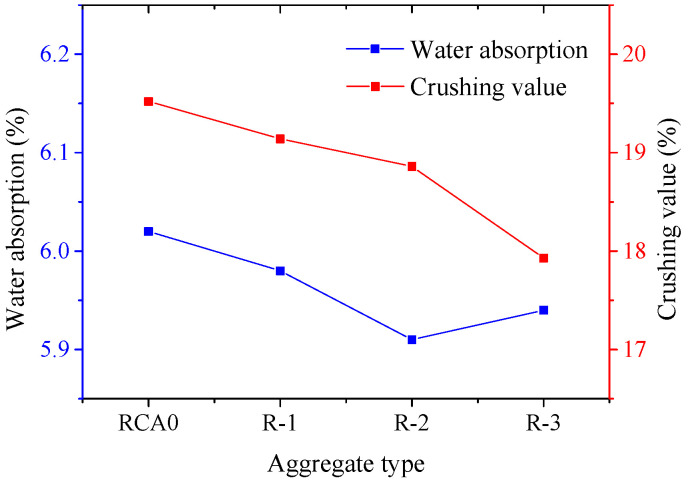
The effects of surface reinforcement on water absorption and crushing value of RCA.

The modified RCA obtained from S-0, S-FA10, and S-GBFS10 pastes were labeled R-1, R-2, and R-3, respectively. Figure 6 displays photographs of the modified RCA and untreated RCA0, with a coin diameter of approximately 25 mm. The water absorption and crushing values of RCA before and after the surface reinforcement are shown in Figure 7. The means of the surface-reinforced RCA were observed to be reduced compared to RCA0 in terms of water adsorption ratios and crushing values. Moreover, Figure 7 shows that R-2 and R-3 exhibited a more significant reduction than R-1 in consistency with the analysis result of strengthening paste properties. However, the improvement in water absorption was not salient, with a maximum decrease of 1.8% in comparison to RCA0. Moreover, the chief reasons for this involved the inability of coated pastes to prevent water from infiltrating and suffusing RCA0 due to the almost negligible amount of the paste compared to the weight of RCA [43]. The result obtained was in agreement with the previous study [28].

To verify the validity of surface reinforcement, the RAC was prepared from RCA0, R-1, R-2, and R-3, and labeled as C-0, C-1, C-2, and C-3, respectively. As the properties of the concrete exhibited a strong correlation with the moisture state of the aggregate, RCA0, R-1, R-2, and R-3 were dried at above 40 °C for 3 days prior to preparing the concrete. The moisture content of RCA0, R-1, R-2, and R-3 were tested to be 1.2%, 1.2%, 1.3%, and 1.1%, respectively. Moreover, the macroscopic properties of RACs, as well as the microstructural properties of ITZs between the MPC and RCA0 or fresh mortar, were investigated.

### 3.3. Macroscopic Properties of Concrete

#### 3.3.1. Mechanical Properties

**Figure 8:**
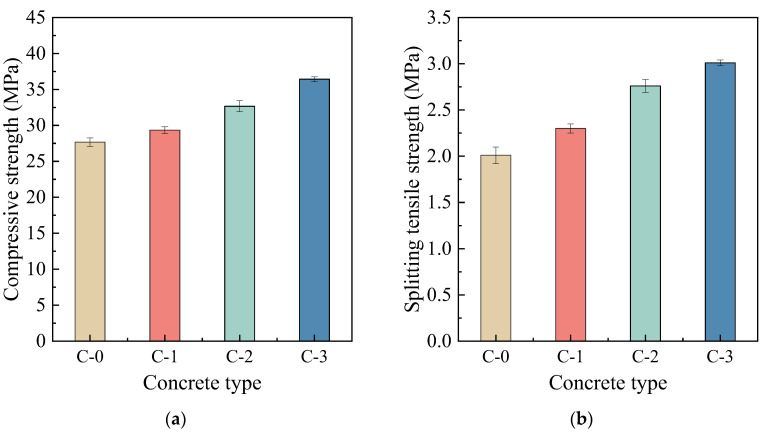
(**a**) Compressive strengths and (**b**) splitting tensile strengths of various concrete.

The 28-day compressive and splitting tensile strengths of all RAC are shown in Figure 8a,b, respectively. It can be seen that the RAC samples obtained after the enhancement treatment with different MPC pastes exhibited increased compressive and splitting tensile strengths compared to C-0. The increase in strengths may be attributed to the high bonding performance of the MPC paste to RCA0 or the new mortar, the filling of micro-defects in RCA0, and the strengthening of the new ITZ. This inference will be substantiated in the following sections. On the condition that 10% FA or GBFS was added, the compressive strength of RAC increased compared to C-1, with increments of 11.32% and 24.13%, respectively. Moreover, C-3 exhibited the highest improvement degree. The trend in splitting tensile strength aligned with compressive strength, and C-1, C-2, and C-3 showed progressive increasing values, which rose by 14.43%, 37.31%, and 49.75%, respectively, compared to C-0. The modified RAC’s mechanical strength variations aligned with the performance of the MPC paste.

#### 3.3.2. Chloride Ion Penetration Resistance

**Figure 9:**
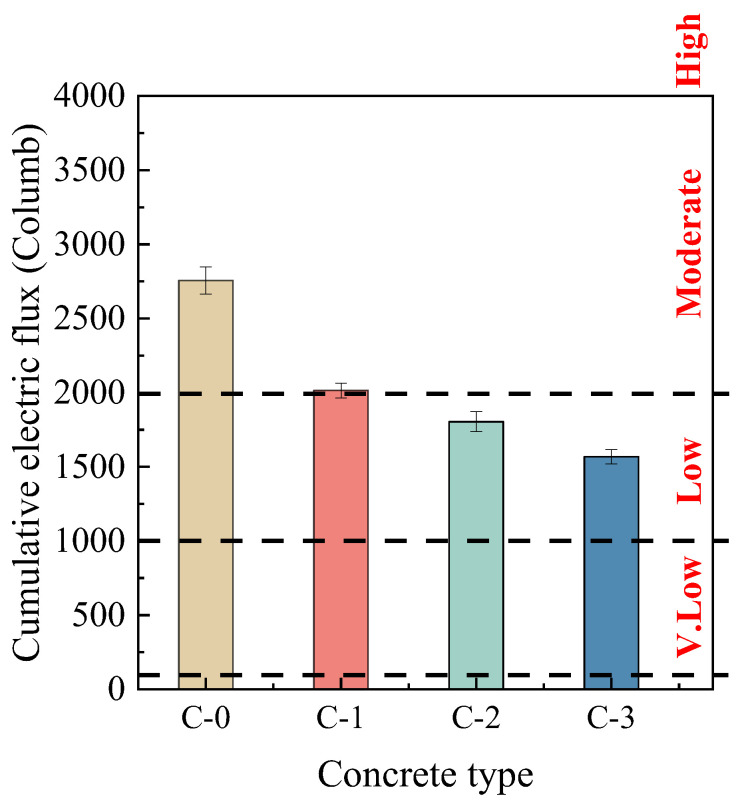
Chloride ion penetration resistances of various concrete.

Figure 9 displays the cumulative electrical flux for each target concrete. As observed in the figure, the precoating for RCA0 with various MPC pastes improved RAC’s chloride ion penetration resistance to varying degrees, possibly due to the enhanced bonding of RCA0 to the new mortar as well as the better chloride ion penetration resistance of the MPC pastes. Detailed evidence for this hypothesis will be elaborated in the following sections. Additionally, double mixing had a favorable effect on the performance of the new ITZ in the modified RAC, thereby improving its chloride ion penetration resistance to some extent. The cumulative electric fluxes passed through C-1, C-2, and C-3 were 2015 C, 1804 C, and 1568 C, respectively, showing a decreasing trend. Compared to C-0, these values represented a reduction of approximately 26.9%, 34.5%, and 43.1%, respectively. The observed variation in RAC’s chloride ion penetration resistance likewise aligned with the performance trend of MPC paste. According to ASTM C1202, untreated C-0 can be classified as “moderate”, whereas the RAC enhanced with S-FA10 or S-GBFS10 was classified as “low”. The reduced chloride ion permeability indicated that modifying RAC with MPC enhanced its anticipated durability.

### 3.4. Interfacial Bond Behavior and Microscopic Characteristics

**Figure 10:**
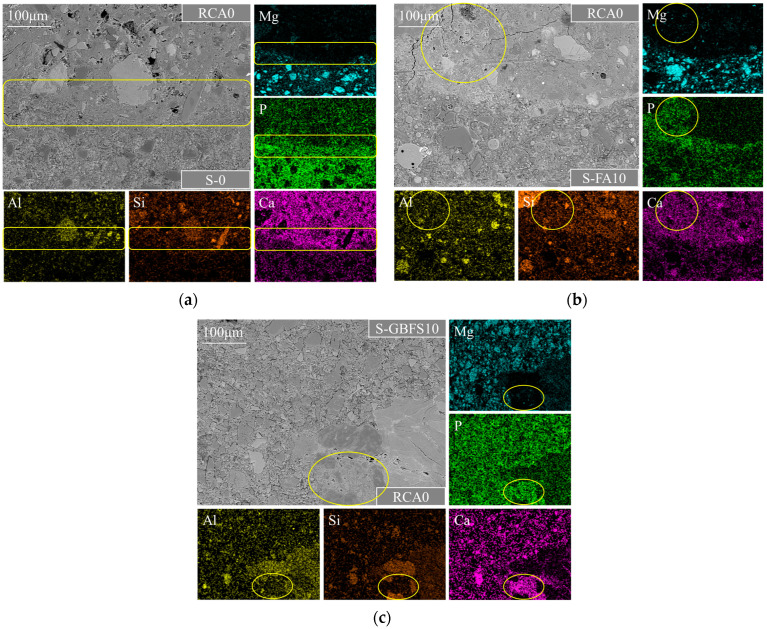
Typical BSE images and elemental maps of the bond interfaces in (**a**) C-1, (**b**) C-2, and (**c**) C-3.

To investigate the bond efficiency of the strengthening paste as a “bridge” and the mechanisms of filling in RCA0 and strengthening the new ITZ, BSE-EDS images were captured at a magnification of 500 times to analyze the microstructure at the interface between various MPC pastes and the new or old mortar. Figure 10a–c presents the typical BSE-EDS images of the bond interfaces between hardened S-0, S-FA10, or S-GBFS10 pastes and RCA0. The images show that all strengthening pastes exhibited excellent bonding with RCA0, forming relatively dense, robust, and uniform interface regions. The regions highlighted with the yellow coils in Figure 10 demonstrated that the P element was prominently incorporated into the old mortar zones, overlapping with the Ca element. This observation indicated excellent mechanical and chemical interlocking ascribed to MPC pastes’ infiltration and filling in RCA0, as well as the reaction between soluble acidic phosphates from the infiltrated MPC pastes and Ca(OH)_2_ in the old mortar, resulting in a favorable bond between the MPC paste and the RCA0. The characteristics mentioned above likewise aided in improving the pore structure of the RCA0 surface, resulting in surface reinforcement. Moreover, mineral admixtures in MPC pastes can operate as fillers through penetrating the pores of the old mortar and interface, as shown in Figure 10b. They might also have a pozzolanic effect that formed new hydrated products and improved the homogeneity and density of the old mortar and interface [69,70].

**Figure 11:**
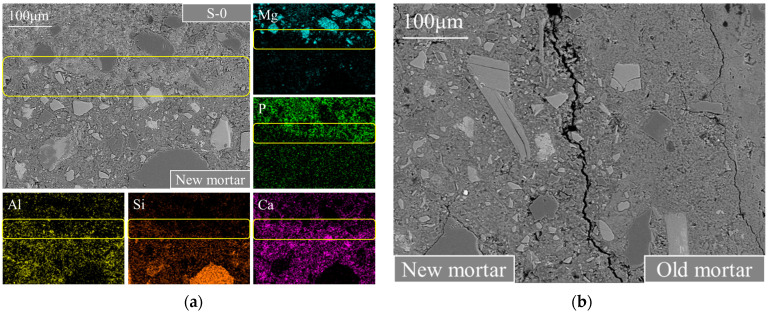
Typical new ITZs’ (a) BSE-EDS image in C-1 and (b) BSE image in C-0.

For preventing interference from elements present in mineral admixtures, C-1 was used as an example to clarify the bonding mechanism of the strengthening paste to the new mortar, as well as its reinforcing mechanism on the new ITZ based on BSE-EDS analysis, as displayed in Figure 11a. For comparison, a typical BSE image of the new ITZ between RCA0 and the new mortar in the untreated C-0 sample is shown in Figure 11b. As seen in Figure 11b, it was evident that there were sizeable cracks in C-0’s new ITZ, probably due to interfacial debonding. Large pores can also be observed within the new ITZ due to the wall effect and increased moisture content. Consequently, C-0’s final performance was significantly weakened. Figure 11a shows a significant reduction of pores and microcracks in the new ITZ of C-1 compared to C-0. The distributions of Ca, P, and Mg elements within the area circled in yellow in Figure 11a indicated that Ca ions from the new mortar permeated into the MPC matrix near the interface, reacting with struvite or unhydrated phosphates to generate new cementitious materials, leading to a denser hardened MPC matrix near the interface and promoting hydration reactions in the new ITZ. The same phenomenon can also be observed in C-2 and C-3. These findings indicated that the MPC pastes exhibited excellent chemical bonding with the new mortar, significantly lowering the likelihood of shrinkage-induced debonding cracks and the appearance of large pores in the modified RAC’s new ITZ. This contributed to the new ITZ’s more compact and superior microstructure, further enhanced through the beneficial effects of the double mixing procedure.

Overall, the MPC paste precoating treatment yielded excellent interfacial bonding properties and microstructure, which exposed the mechanism for obtaining improved mechanical strengths and chloride ion permeability resistance of the modified RAC. Meanwhile, these findings also provided evidence to support the inferences in Section 3.3.

### 3.5. ITZ Microhardness Analysis

**Figure 12:**
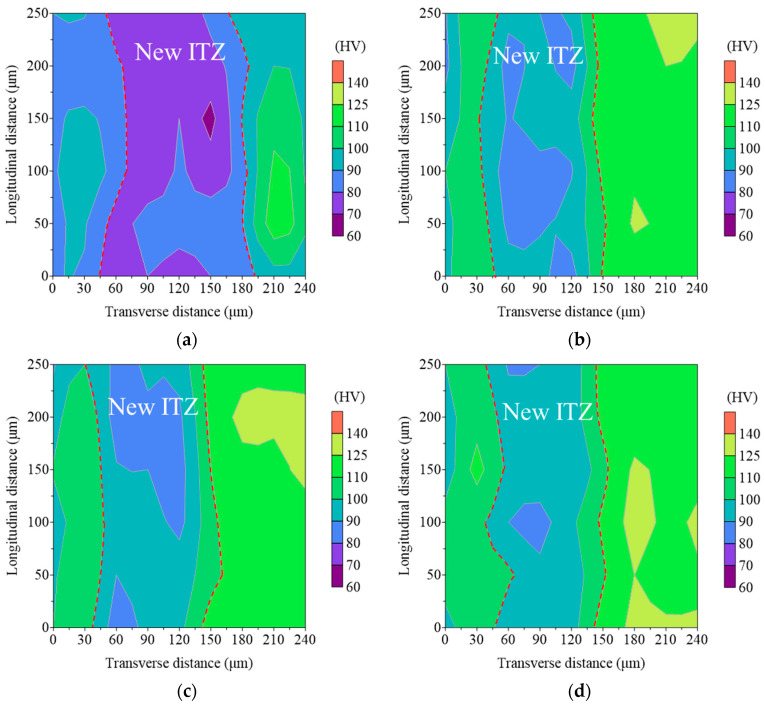
Typical microhardness distribution maps within indent areas of (**a**) C-0, (**b**) C-1, (**c**) C-2, and (**d**) C-3.

**Figure 13:**
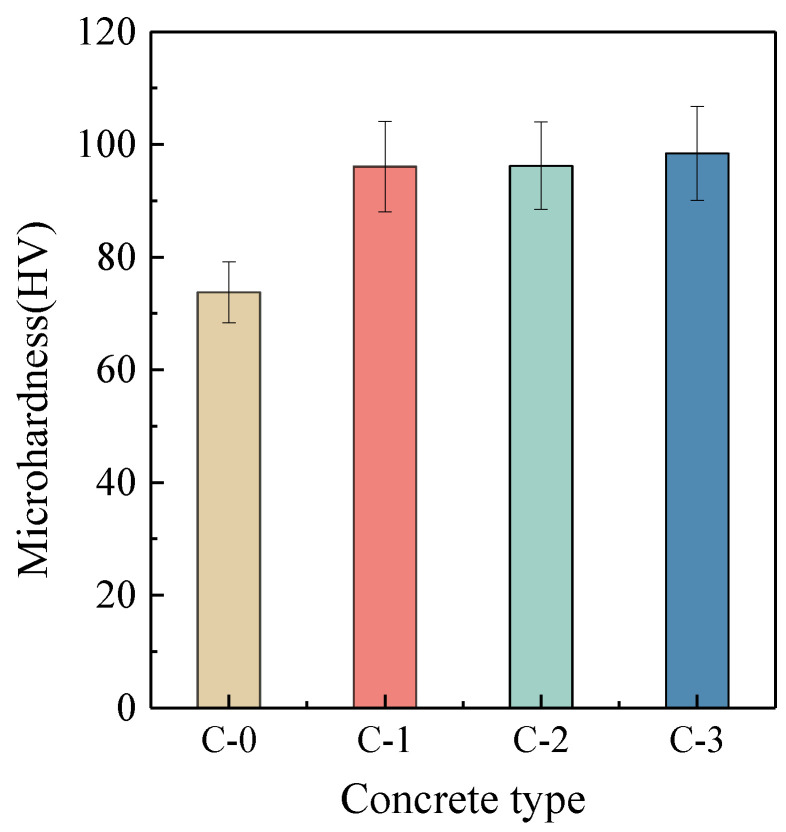
The average microhardness values of the new ITZs in C-0, C-1, C-2, and C-3.

Microhardness analysis was used to quantitatively evaluate the micro-mechanical characteristics of the new ITZ in RAC to define the improving effectiveness of the surface treatment method employing MPC paste. Figure 12 and Figure 13 present the typical microhardness distribution maps and the average microhardness values for the new ITZs of C-0, C-1, C-2, and C-3. The boundaries of the new ITZ in the microhardness distribution maps were depicted with red dashed lines. In all samples, the microhardness values were relatively low when located within the ITZs (with a width of approximately 85 μm to 150 μm), but they rose and stayed steady as one moved away from the ITZs, as shown in Figure 12. Figure 12a–d shows that the new ITZs’ widths (approximately between 85 μm and 110 μm) following enhancement with various MPC pastes dramatically decreased in comparison to the width (approximately between 110 μm and 150 μm) of the untreated C-0, accompanied with higher microhardness values. The average microhardness values of the new ITZs in C-1, C-2, and C-3 rose by 30.2%, 30.4%, and 33.4%, respectively, compared to C-0, as shown in Figure 13. These findings indicated that the new ITZ of the modified RAC has been effectively strengthened, aligning with the BSE observations and providing further evidence for the effectiveness of the proposed strengthening method in this study.

## 4. Conclusions

In this work, seven surface-strengthening pastes were prepared; then, compressive strength and chloride ion penetration resistance were comparatively studied; afterward, the most suitable FA–MPC and GBFS–MPC hybrid slurries with the best comprehensive performance were used to coat RCA0, followed by 3 days of maintenance, and MPC slurries without mineral admixtures were also selected for comparison purposes; lastly, the physical properties of RCA before and after the surface reinforcement were compared, and the macroscopic and microscopic properties of target concrete were evaluated. The conclusions are summarized as follows:

(1) S-FA10 and S-GBFS10 were most suitable to coat RCA0 due to the higher strength and chloride ion penetration resistance, and the reason was that the mineral admixtures facilitated secondary reactions and enhanced the integrity of the hardened pastes.
(2) After the surface reinforcement with S-0, S-FA10, and S-GBFS10, the crushing value of RCA decreased from 19.52% to 19.14%, 18.86%, and 17.93%, respectively. Nevertheless, surface-strengthening pastes had little effect on the water absorption of RCA. Furthermore, the RAC prepared from R-1, R-2, and R-3 performed better than that from RCA0 regarding mechanical properties and durability. The enhancement efficiencies on the performance of RCA and RAC improved with strengthening paste performance.
(3) The BSE-EDS observations of the modified RAC showed the presence of mechanical and chemical interlocking between the strengthening paste and RCA0 or new mortar, which led to the effective filling of micro-defects near the RCA0 surface and the well-bonded interfaces between MPC pastes and RCA0 or new mortar. Furthermore, a denser microstructure within the new ITZ was observed to further improve the strengths and durability of the RAC under the combined effect of the precoating treatment and the double mixing method.
(4) Based on the microhardness test results of the ITZs, it can be seen that the breadths of the new ITZs were reduced, and the average microhardness values were improved after modification with MPC pastes, showing an improvement in its micromechanical properties, which further confirmed the effectiveness of the surface-strengthening treatment using MPC pastes.

Overall, the surface treatment method proposed in this study was considered effective and applicable. Furthermore, the construction duration was shortened compared to previous studies [34], thus improving the operability of RCA for applications in practice. In the future, the effects of MPC slurry precoating treatment on the morphology of RCA [71] as well as on heat resistance [72] and the internal moisture content [73] of RAC need further investigation, which has essential implications for the application of MPC in the surface modification of RCA.

## Figures and Tables

**Table 1:** Chemical composition of raw materials (by wt/%).

Raw Materials	Mass Fraction of the Sample (%)									
	SiO_2_	Al_2_O_3_	CaO	Fe_2_O_3_	K_2_O	TiO_2_	Na_2_O	SO_3_	MgO	P_2_O_5_
FA	49.80	30.69	5.30	5.08	2.23	2.02	1.54	1.25	1.11	0.47
GBFS	35.51	13.11	39.82	0.37	0.29	2.63	0.37	2.26	4.88	0.02
MgO	2.35	1.30	1.31	1.27	0.02	0.03	0.04	-	92.12	0.12

**Table 2:** Properties of Portland cement.

Cement Type	Density (kg/m^3^)	Specific Surface Area (m^2^/kg)	Setting Time (min)		Compressive Strength (MPa)		Flexural Strength (MPa)	
			Initial Setting	Final Setting	3 d	28 d	3 d	28 d
OPC	3090	398	220	310	25.1	47.3	4.5	7.9

**Table 3:** Materials ratios of MPC pastes for modifying 1000 kg RCA0.

Paste Type	ADP (kg)	MgO (kg)	FA (kg)	GBFS (kg)	Water (kg)	B (kg)
S-0	93.2	186.4	0	0	50.9	8.4
S-FA5	93.2	177.08	9.32	0	50.9	8.4
S-FA10	93.2	167.76	18.64	0	50.9	8.4
S-FA15	93.2	158.44	27.96	0	50.9	8.4
S-GBFS5	93.2	177.08	0	9.32	50.9	8.4
S-GBFS10	93.2	167.76	0	18.64	50.9	8.4
S-GBFS15	93.2	158.44	0	27.96	50.9	8.4

Note: S-0 denotes strengthening slurry without mineral admixture; “S-FA” and “S-GBFS” denote strengthening slurries with FA and GBFS, respectively; the numbers “5”, “10”, and “15” denote the percentage of MgO replaced with mineral admixture.

## Data Availability

The data presented in this study are available on request from the corresponding author.
